# A single nucleotide variant of human PARP1 determines response to PARP inhibitors

**DOI:** 10.1038/s41698-020-0113-2

**Published:** 2020-04-27

**Authors:** Rivki Cashman, Alona Zilberberg, Avner Priel, Hagit Philip, Alexander Varvak, Avi Jacob, Irit Shoval, Sol Efroni

**Affiliations:** The Mina and Everard Goodman Faculty of Life Sciences, Bar Ilan University, Ramat-Gan, 52900 Israel

**Keywords:** Cancer therapy, Cancer genomics, Predictive markers

## Abstract

The introduction of novel cancer drugs and innovative treatments brings great hope for cancer patients, but also an urgent need to match drugs to suitable patients, since certain drugs that benefit one patient may actually harm others. The newly developed poly-ADP ribose polymerase (PARP) inhibitors (PARPis) are a group of pharmacological enzyme inhibitors used clinically for multiple indications. Several forms of cancer tend to be PARP dependent, making PARP an attractive target for cancer therapy. Specifically, PARPis are commonly used in BRCA-associated breast cancers patients, since unrepaired single-strand breaks are converted into double-strand breaks and BRCA-associated tumors cannot repair them by homologous recombination so that PARPi leads to tumor cell death, by a mechanism called “Synthetic Lethality”. Unfortunately, not all patients respond to PARPi, and it is not currently possible to predict who will or will not respond. Here, we present a specific genomic marker, which reflects a single-nucleotide polymorphism of human PARP1 and correlates in vitro with response to PARPi, throughout all indications. In addition, we report that this SNP is associated with re-shaping mRNA, and mRNA levels, and influences the final protein structure to expose new binding sites while hiding others. The status of the SNP is therefore critical to patients’ care, as it relates responses to PARPi to the PARP1-SNP carried.

## Introduction

Poly-ADP ribose polymerase (PARP) inhibitors (PARPis) are a group of pharmacological inhibitors of PARP that have been developed clinically for multiple indications. Several forms of cancer are dependent on PARP, making the molecule an attractive target for cancer therapy^1^. As an example, PARPis are commonly used in BRCA-associated breast cancers patients since unrepaired single-strand breaks are converted into double-strand breaks (DSBs) and the BRCA-associated tumors cannot repair them by homologous recombination, so that PARPi leads to tumor cell death. Initial studies showing that PARP1 inhibition resulted in synthetic lethality in BRCA1 and BRCA2-deficient cell lines^2,3^, and the preclinical studies that followed^4,5^, paved the way for clinical studies, which have recently reported major successes in treatment. Unfortunately, only around 30% of patients would show overall response to PARPi treatment. Moreover, it is currently not possible to associate a patient with the response groups^6–8^.

There are multiple factors that are known to influence the efficiency of PARPi, including HR and non-homologs-end-joining (NHEJ) status, PARP1 levels or its activity, and other factors that modulate the intracellular concentration of PARPi^9^. It is therefore necessary to assess the status of these controlling factors before initiating PARPi treatment^10,11^. In addition, a thorough understanding of the various mechanisms responsible for resistance to PARPi may facilitate the design of improved PARPi mono and combination therapy, and identify ways to re-sensitize tumor cells to PARPi, and treat cancer patients more efficiently^9^.

As already described, the drug response to PARPis is not uniform across patients—a treatment that is beneficial for some people may be ineffective, or even fatal, for others. We^12^ and others^13^ have shown that it may be possible to match drug response to patients’ germline sequence, through the status of specific genomic polymorphisms.

A single-nucleotide polymorphism (SNP) is a single base-pair variant of a specific site in the germline DNA sequence. To classify a variation as an SNP, it should occur in at least 1% of the population^14^. SNPs are responsible for most of the variation between any two individuals and are known to be associated with specific predispositions to diseases, and drug metabolism, among other properties^15^.

The central dogma of molecular biology holds that, since synonymous SNPs do not change the coded amino acids, they are therefore not expected to change the function of the produced protein. However, it has now been demonstrated that an SNP can affect protein conformation and function, leading to altered disease susceptibilities, differential prognosis, and/or drug responses, among other clinically relevant genetic traits^13^.

The evidence that a synonymous SNP can play a role in influencing protein function is at the core of this work. Here we describe a combination of methods that enable us to identify functional changes in a protein, and correlate them with drug response, in order to provide a biomarker and a possible mechanism for the effect. The results presented here may provide the signature needed to predict a patient response to PARPi treatment and may also provide the conceptual tools required to resolve similar issues for other novel drugs or novel combinations.

## Results

### A single, synonymous, nucleotide variant of the PARP1 sequence determines the levels of RNA expression

**Fig. 1:**
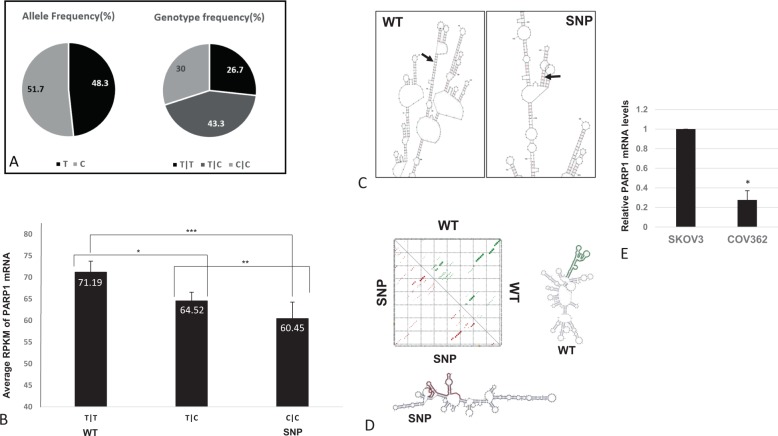
The PARP1 variant: frequency in population; RNA expression levels in patients and the transcripts’ secondary structure. **a** rs1805414 allele and genotype frequencies from the “1000 genome” project. **b** PARP1 RNAseq levels from 415 patients with breast cancer were obtained from the TCGA and analyzed according to their rs1805414 genotype status. The *Y*-axis represents averaged RPKM levels, and the *X*-axis represents the SNP genotype. *P* values were calculated using unpaired two-tailed *t*-test (*p = 0.0409; **p = 0.345; ***p = 0.0194). **c** The secondary structure of WT, SNP variant PARP1, was determined using predicted free energies via thermodynamic methods. The location of the variant nucleotide is indicated by an arrow for each PARP1 polymorphism. Analysis was conducted by mFOLD. **d** We used RNAsnp to produce Dotplots and to show base-pair probabilities corresponding to changes in global regions. A major change in structure associated with change in sequence was determined by the RNAsnp tool between both variants. WT PARP1 represented in green while SNP variant in red. **e** The Ovarian cell lines COV362 (SNP/SNP) and SKOV3 (WT/WT) were harvested and extracted for their total RNA. mRNA levels of endogenous PARP1 were determined by qPCR as compare to β-actin (*p = 0.016).

The variant rs1805414 is located at chr1:226385663 (GRCh38), with nearly an equal distribution of T and C (see Fig. 1a for frequencies). For the sake of brevity, we will use here the following terminology: the GCC human PARP1 will be referred to as “SNP” and the GCT human PARP1 variant will be referred to as “WT”. We looked for an association between the status of the variant and PARP1 gene expression by mining TCGA (http://cancergenome.nih.gov/abouttcga) gene expression data from 415 patients with breast cancer. Interestingly, the results (Fig. 1b) revealed significantly lower levels of PARP1 expression in patients with the SNP version of PARP1.

A possible explanation for a relationship between sequence variants and gene expression is the influence that specific variants may have over mRNA secondary structure and thereby on transcript stability and degradation^16^. In order to investigate a possible connection between the sequence variation and the secondary structure of PARP1, we used the RNA folding algorithms mFOLD^16,17^ and RNAsnp^18^. The output obtained from mFold (Fig. 1c) indicated that this specific variant may affect the secondary structure. RNAsnp allows an investigation of the specifics of the secondary structure options that are produced by the WT and SNP variants of PARP1. In Fig. 1d, the structural regions most influenced by this sequence change are colored according to *p* value where *p* values greater than 0.2 (i.e insignificant structural changes) are in black. The highlighted values in the figure correspond to *p* values of 0.1157.

To study RNA levels in cells, we used two ovarian cancer tumor cell lines, where SKOV3 harbors the WT PARP1 variant and COV362, the SNP PARP1 variant. The presence of these sequence variants was confirmed first through the Broad Institute Cancer Cell Line Encyclopedia, CCLE^19^ and then, using Sanger sequencing, with a set of primers directed to contain the variable sequence (Supplementary Fig. 1). We harvested mRNA from the two cell lines and quantified the endogenous PARP1 levels using RT-PCR compared to actin levels. Interestingly, the mRNA levels of the SNP sequence of PARP1 (from COV362) were significantly lower than those of the WT sequence (from SKOV3) (see Fig. 1e).

**Fig. 2:**
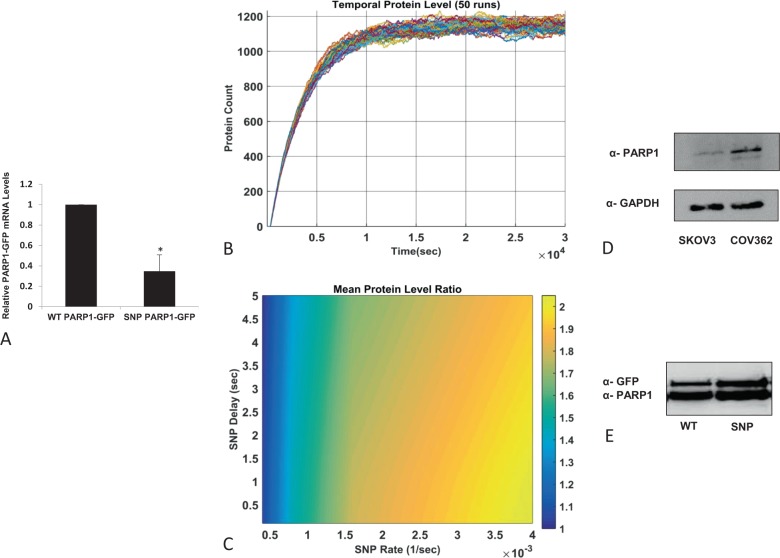
A simulation of ribosome function over the two different PARP1 variants shows the resulting differences in PARP1 protein levels. **a** HEK293T cells were transfected with WT PARP1-GFP or with the SNP PARP1-GFP plasmid. Relative GFP-PARP1 mRNA levels represented the ratio between the SNP variant and WT PARP1, using qPCR. A set of GFP primers were used to determine the overexpressed GFP-PARP1 variants, normalized to endogenous β-actin (*p = 0.004). **b** Protein level in 50 simulated realizations (runs) for particular parameters set. The stochastic nature of the simulation is apparent in the steady-state phase, where the level fluctuates around some mean value. **c** Protein level ratio between SNP and WT parameters. The reference WT parameters were taken to be: *k*_WT_ = 0.4*e*^−3^, *τ*_WT_ = 0.1. **d** COV362 (SNP/SNP) and SKOV3 (WT/WT) were harvested for their protein. Cell lysates were subjected to SDS-PAGE gel and transferred to a nitrocellulose membrane. Anti-PARP1 detected the endogenous levels of PARP1. GAPDH measurement served as a loading control. **e** HEK293T cells were transfected with WT PARP1-GFP or with the SNP PARP1-GFP plasmid. Lysates from each transfection were subjected to SDS-PAGE gel and transferred to a nitrocellulose membrane. Anti-GFP detected the overexpressed levels of GFP-PARP1 variants. Endogenous PARP1 measurement served as a loading control.

To control for the effect of endogenous components on PARP1 expression and to better associate mRNA expression levels with the sequence variant, we introduced the SNP variant rs1805414 into a PARP1-GFP vector, using site-directed mutagenesis. We then overexpressed the vectors in HEK293T cells, and 48 h later, purified total RNA. Quantification of the GFP-PARP1 mRNA transcript normalized to endogenous actin indicated significantly lower levels of the SNP variant (*p* value < 0.004, Fig. 1b), PARP1 mRNA than the WT variant (Fig. 2a).

Every organism has multiple tRNA species that read the codons for the same amino acid (tRNA isoacceptors). Differences in the relative abundance of tRNA isoacceptors have been found to affect the level of highly expressed proteins. This tRNA abundance–codon distribution relationship can have predictive power over the expression of genes based on their codon usages^20^. According to the Codon Usage Database of Homo sapiens^21^, the frequency ratio of GCC/GCT is 1.5. As the next stage, we introduced an artificial variant of PARP1 with GCG (frequency ratio GCT/GCG of 2.48), and overexpressed it in the same fashion. In this case, the levels of mRNA levels of the GCG PARP1 variant were higher than those of the WT PARP1-GFP (Supplementary Fig. 2).

### Simulating the effect of the ribosomes on PARP1 function in the context of a silent variant

To investigate one possible mechanism by which a silent variation may influence the folded protein, we computationally studied the effect codon changes may have on ribosome function (simulation illustration; Supplementary Fig. 3). It is generally accepted that the speed at which ribosomes decode codons depends on the cellular availability of the tRNA that matches the codon^22^. The most abundant codons pair with the most abundant matching tRNAs and vice versa^23^. We propose that synonymous single variants, which require relatively low or relatively highly abundant tRNAs, may influence translation rates. Thus, the paucity of a specific tRNA may result in (1) inhibition of the ribosome complex, (2) mRNA accumulation, and (3) changes in conformation and quantity of the mature protein. We can simulate the first event in the cascade, ribosome inhibition, by modeling its dynamics (Fig. 2b, c). Reactions at the single-cell level and at low copy numbers are inherently stochastic. Conventional deterministic models are unable to capture this behavior; hence, stochastic simulation algorithms were developed. The principle method for stochastic simulations is the Gillespie Algorithm, first presented in 1976 (ref. ^24^), which simulates the Markov processes behind the model. The system is described by its state vector, which contains the copy numbers of each species, and the parameters for each reaction, e.g., the probability that a particular reaction will occur. For typical eukaryotic cells, the translation of an average protein takes about 450 s. There is therefore a delay between the initiation and completion of the reaction, which should be incorporated into the model^25^. For this purpose, we use the delayed Stochastic Simulation Algorithm (delayed SSA)^26^, which encompasses a delay in the release of reaction products following the initiation of an event. This is done by storing products in a waitlist and releasing them after the proper time has elapsed. We have developed a model designed to describe the translation process at the single-cell level with particular focus on the variable (SNP) codon. The model considers a small population of ribosomes translating either WT or SNP-mRNA and provides a crude description of the pre/post sub-processes, i.e., the translation of the subsequence before the particular codon, as well as the subsequence after it. This breakdown allows us to investigate the possible contribution of the codon translation under different reaction conditions, and in particular its rate and time delay. The simulation results were in accordance with the experimental observation that variability in the SNP reaction rate gives rise to a considerable difference in the average amount of protein produced. The system described above was simulated using the tool “SGNsim” (version 2)^27^.

Figure 2b shows typical runs of the simulation system for a given set of parameters.

Each set of parameters was run 50 times in order to analyze the results statistically, where the variable of interest is the time-dependent average protein level, measured after the transient time of simulation, i.e. $t \,>\, 10^4\,{\mathrm s}$ (see Fig. 2b). We do not compare the simulation results with the absolute values observed experimentally but instead are interested in the relative behavior of the WT vs. the SNP mutation. For this reason, we assume typical values for the parameters in the case of the WT in Eq. (2), *k*_WT_ = 0.4 × 10^−3^ s^−1^ and *τ*_WT_ = 0.1 s, based on the review^26^.

Figure 2c presents the ratio of protein levels, taken at post transient time, for various combinations of the two variables, SNP delay (*τ*_snp_) and SNP rate (*k*_snp_), and the values for the WT. At values of SNP rate *k*_snp_ > 10^−1^, the SNP exhibits a more than 50% increase in the protein level. We note that the delay parameter seems to be less effective.

### Sequence variation mediates levels of the mature protein

As the simulation demonstrates, ribosome inhibition, caused by a shortage in tRNA supply, leads to higher levels of mRNA transcript and a decrease in protein levels. In contrast, a continuous supply of tRNA ensures high translation rates and leads to higher levels of protein. In order to verify these findings experimentally, we purified total protein from both SKOV3 (WT version of PARP1) and from COV362 (SNP version of PARP1) cells. Endogenous PARP1 was assayed using anti-PARP1 antibody. In agreement with the simulation results, the SNP variant PARP1, in the COV362 cell line, showed higher levels of protein (Fig. 2d) with lower levels of mRNA (Fig. 1e), while the SKOV3 cell line, with the WT version of PARP1, showed lower levels of the protein.

The tRNA usage argument for determining protein levels can also be tested by using PARP1-GFP variants. HEK293T cells were transfected with each of the two PARP1 variants coupled to GFP. As can be seen in Fig. 2e, cells transfected with the SNP PARP1-GFP variant expressed relatively higher levels of protein than those transfected with the WT PARP1-GFP. All together, these results indicate that substitution of a single synonymous nucleotide at the position Chr 1: 226385663 (GRCh38) leads to variation in the levels of expressed protein.

### A single synonymous variation may modify protein structure–function

We further speculated that ribosome inhibition could also intervene/interfere with conformation of the newly synthesized peptide. Our hypothesis is that changes in the rate of activity of a ribosome could have implications for the structure of the mature protein if ribosome delay (due to low tRNA availability) leads to temporal and local folding of the newly synthesized peptide. Subsequently, these local variations in folding could affect binding to other proteins or alter the exposure/concealment of post-translation modification sites, thereby changing the function of the protein.

**Fig. 3:**
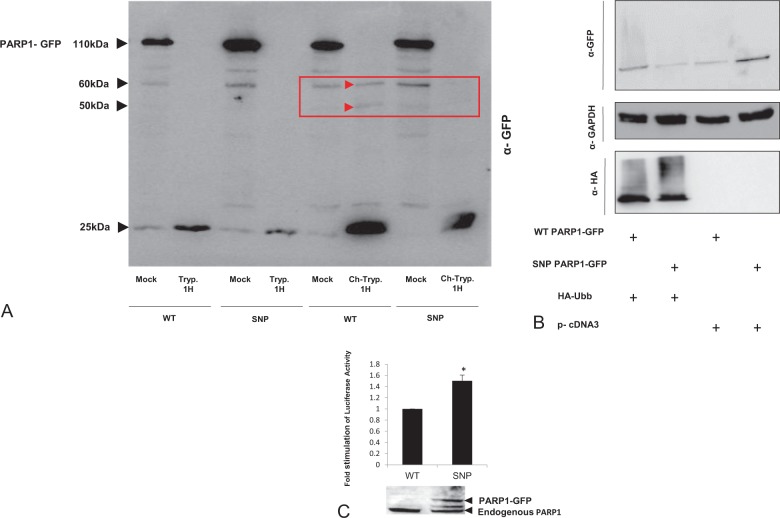
Sequence variation in PARP1 could lead to two different protein structures. **a** HEK293T cells were transfected with WT PARP1 GFP or SNP PARP1 GFP for 48 h incubation. Cells were harvested and lysates were treated at the presence of Trypsin or Chymotrypsin to induce a controlled enzymatic cleavage for 1 h, at 37 °C. The cleaved lysates were subjected to SDS-PAGE gel followed by blotting to membrane and incubation with anti-GFP. Red arrows indicating two bands on the WT-PARP1 which are absent on the SNP variant. **b** PARP1 WT or SNP-GFP were transiently co-transfected to HEK293T cells along with UBB-HA or empty vector. Forty-eight hours later, cells were harvested and subjected to SDS-PAGE gel. By using anti-GFP antibody we detected the overexpressed GFP-PARP1 of both the WT version and the SNP variant. The un-bounded UBB-HA was detected by anti-HA antibody. GAPDH measurement served as a loading control. **c** 293T cells were co-transfected with GFP PAPR1 WT or SNP, b-gal, and TOP-FLASH. Forty-eight hours post transfection, cells were harvested and monitored for their Luciferase levels, normalized to b-gal values. (*p = 0.04). The same lysates were subjected to SDS-PAGE gel to verify the endogenous levels of PARP1 compare to the exogenous levels of WT and SNP PARP1 GFP variant using the anti-PARP1 rabbit antibody.

The mature proteins obtained from different gene sequence templates may therefore vary somewhat in the secondary, tertiary, and quaternary structures, in comparison to the WT template. The outcome may have considerable ramifications in that such changes could re-shape the interactions of a protein with its endogenous components or with drugs targeting the protein. In order to confirm that synonymous changes may alter protein structure, we established an in vitro cleavage assay. HEK293T cells were transfected with either the WT or SNP version of PARP1-GFP. After 48 h incubation, the cells were lysed and incubated for 1 h with trypsin or chymotrypsin to obtain a controlled enzymatic cleavage. Lysates were subjected to SDS-PAGE gel and blotted proteins were incubated with anti-GFP antibody. As can be seen from the blot (Fig. 3a), trypsin cleavage produced the same digestion pattern from both WT and SNP versions of PARP1-GFP: a one-step digestion with accumulation of the 25 kDa C′-terminal-tagged GFP product. In contrast, the 1 h incubation with chymotrypsin produced a different digestion pattern across PARP1-GFP variants (Fig. 3a): while the SNP version PARP1-GFP substrate was cleaved to generate a 25 kDa GFP-tagged product, there were two additional products of digestion of the WT- PARP1-GFP (50 and 60 kDa), as indicated by the arrows in Fig. 3a, and a third final product of 25 kDa.

Based on these results, we sought to identify additional topological variations with potential mechanistic implications that could also result from the two templates. For this purpose, we designed a ubiquitin assay, employing construct pcDNA3-HA-UBB. HEK293T cells were co-transfected with the SNP or WT versions of PARP1 GFP and with HA-ubiquitin (UBB-HA) or pcDNA3. The results showed that UBB-HA not only enhanced the expression levels of the WT version PARP1 GFP compared to the SNP variant (Fig. 3b) but also induced post-translation modification of the WT version PARP1 GFP as seen by the smear accompanying the WT version PARP1 GFP band. Such patterns are typical of post-translational modifications monitored by western blot analysis and indeed the extent of the smear was correlated with the addition of overexpressed UBB. As expected, co-transfection of the WT version PARP1 GFP with vector produced only limited expression as compared with the SNP variant.

In order to investigate whether any functional changes are driven by this specific polymorphism, we examined the role of PARP1 in Wnt signaling. PARP1 is one of the co-activators of the transcription complex TCF-4/β-catenin. During apoptosis, cleavage of PARP1 by caspase-3 (ref. ^28^) is likely to affect the interaction between PARP1 and TCF-4 (ref. ^29^), and terminate Wnt signaling.

To study the effects of PARP1 variants on Wnt signaling, we used a Luciferase reporter assay based on a construct containing three copies of the wild-type TCF-4-binding element (pTOP FLASH), inserted upstream to the Luciferase gene. Cells were co-transfected with β-catenin, β-gal, pTOP FLASH, and WT PARP1 GFP or SNP PARP1 GFP. Wnt signaling rate was assessed by the Luciferase assay system (Promega) and the arbitrary luciferase values were normalized to β-galactosidase levels, in each transfection. As presented in Fig. 3c, transfection with SNP PARP1 GFP dramatically increased Wnt signaling (1.5-fold) compared to the WT version. The same lysates were subjected to SDS-PAGE gel to verify the endogenous levels of PARP1 compared to the exogenous levels of WT and SNP PARP1 GFP variant. As expected, the SNP PARP1 GFP variant, which expressed higher protein levels than the WT version of PARP1, also induced higher levels of Luciferase.

### PARP1 variants may differ in response to PARP1i

Drugs targeted to a given proteins are usually designed to interact with specific binding domains of the protein of choice. It was therefore of great interest to verify whether the structural or topological changes induced by substitution of a single nucleotide, as demonstrated in Fig. 3, affected the interaction of the protein with PARP1 inhibitors.

**Fig. 4:**
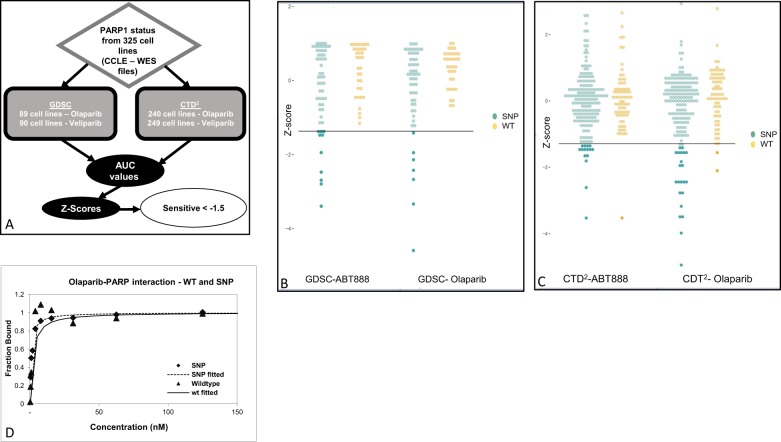
The two PARP1 variants may lead to different responses to PARP1i. **a** Schematic presentation of data mining procedure of the GDSC and CTD2 reservoirs, in aid of CCLE WES files in regard to PARP1 status across cell lines. Response rate for Olaparib and Veliparib were measured in two different cell line datasets—GDSC (**b**) and CTD2 (**c**). For each cell line the AUC value was measured, and a *Z*-score was calculated. The cell lines were separated according to their PARP1 status. The black line separates the sensitive cell lines (*Z*-score < −1.5) and the non-sensitive (*Z*-score > −1.5). **d** HEK293T cells were transfected with GFP-PARP1 WT or SNP. Forty-eight hours later, cells were lysed in HEPES Biacore buffer (10 mM HEPES, 150 mM NaCl, 3 mM EDTA, 0.05% TWEEN-20) and protein inhibitor cocktail (Sigma Aldrich Co.). Series S Sensor chip CM5 single (GE) was pre-treated with Trap-GFP antibody (ChromoTek GmbH, gt-250). Each lysate was streamed through the different chip channels, attached by the Trap-GFP antibody and further incubated with 13 serial dilutions of PARP1 inhibitor Olaparib (LKT-O4402) (starting con. 250 nM). The sensogram is presented, as obtained from Bicore T100 analysis. WT PARP1 *K*_D_ = 0.5 nM; SNP PARP1 *K*_D_ = 0.1 nM.

For this purpose, we combined data from two cell line databases: GDSC^30^ and CTD2 (refs. ^31,32^). The PARP1 status of selected cell lines was retrieved from the Cancer Cell Line Encyclopedia (CCLE)^19^, as described in Fig. 4a and the cells were analyzed for their PARP1 status and response to a series of PARP1 inhibitors. Cell lines representing all three PARP1 genotypes, WT/WT, WT/SNP, and SNP/SNP, were selected from the databases, with the WT/SNP cell lines considered as the SNP cohort. We then compared the PARP1 status with the area under the curve (AUC) values obtained in response to the PARP1i drugs Olaparib and Veliparib (ABT-888). The sensitivity to Olaparib was significantly (*p* value = 0.0125 and 0.0167—GDSC and CTD2, respectively) SNP-related, but although the SNP cell lines were more sensitive to Veliparib, the difference from the WT was statistically insignificant (*p* value = 0.7521 and 0.406—GDSC and CTD2, respectively).

Processing the AUC values as the corresponding *Z*-score index confirmed the results. As can be seen in Fig. 4b, c, the SNP-PARP1 group of cells from both databases were very sensitive to Olaparib (cell lines with a *Z*-score below −1.5). The response to Veliparib revealed a similar trend but fewer cell lines had a *Z*-score lower than −1.5. Importantly, all the sensitive cell lines with *Z* scores beneath −1.5 were BRCA1 mutation independent.

Biacore assays evaluate target molecules, most frequently proteins, by immobilizing them on a prepared gold sensor surface. A sample containing a potential interacting partner in solution is then injected over the surface through a series of flow cells. During the course of the interaction, polarized light is directed toward the sensor surface and the angle of minimum intensity reflected light is detected. This angle changes as molecules bind and dissociate and the interaction profile is thus recorded in real time in a sensorgram^33^.

In order to identify additional possible conformational variations in the PARP1 variants, we designed a Biacore T100 binding affinity assay for PARP1-GFP overexpressed variants to a single PARP1 inhibitor. Specifically, we assessed the binding affinity of PARP1 variants by evaluating their *K*_D_ values for Olaparib where higher values of *K*_D_ represent low affinity of PARP1 protein to Olaparib and vice versa. GFP-PARP1 WT or SNP were overexpressed in HEK293T cells and lysates harvested were immobilized on a gold sensor surface chips that had been pre-treated with ChromoTek GFP-Trap® (ChromoTek) and incubated with serial dilutions (×12) of PARP1i Olaparib. The results (Fig. 4d) indicated that Olaparib was more effective on SNP PARP1, which exhibited a lower value of *K*_D_ (0.1 nM) than the WT version of PARP1 (*K*_D_ = 0.5 nM).

**Fig. 5:**
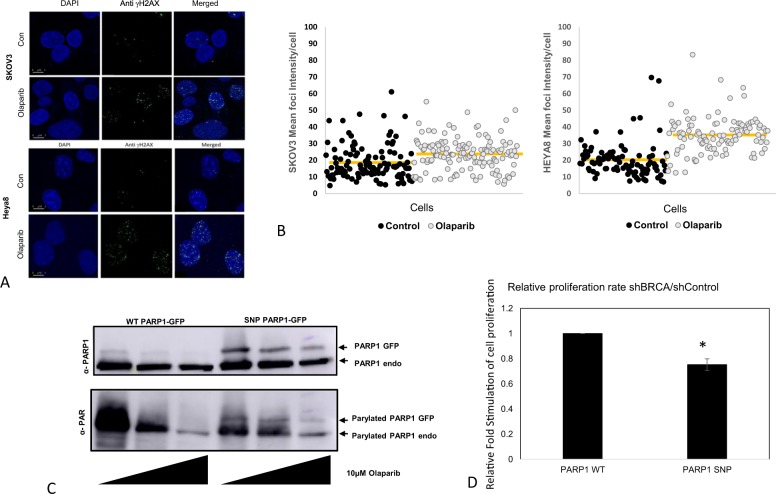
PARP1 variants display diverse intra-modulations which are protein structure dependent. **a** Ovarian cell lines, SKOV3 and Heya8, which harbor endogenous WT-PARP1 and SNP-PARP1, respectively, were incubated with 10 µM Olaparib for 2 h, at 37 °C compare with MOCK cells. Cells were fixed, washed with PBS-Triton X-100 buffer to allow anti-γ-H2AX antibody permeabilization and incubated with a secondary anti-FITC goat anti-rabbit. DAPI staining and mounting were performed. We visualized the typical foci-like pattern by the florescence microscope, Leica confocal live, at ×60 magnitude. Representative scale bars are indicated. **b** Using cell profiler tool we counted the number of foci in each cell line under no treatment (basal phospho-H2AX)—control and under Olaparib incubation. For both cell lines, the mean foci-intensity for each cell was calculated. Yellow line indicates the average intensity for each group (for both SKOV3 and Heya8 *p* < 0.01). **c** HEK293 stably expressed WT-PARP1-GFP or SNP-PARP1-GFP were treated with elevated doses of Olaparib (0, 0.01, and 0.1 µM) for 30′ at 37 °C. Lysates were subjected to SDS-PAGE gel and the blotted membrane was incubated with anti-PARP1 and with anti -GAPDH. The membrane was re-blotted to anti-PAR polyclonal antibody. **d** Stable sh-BRCA1 or sh-con HEK293 cells were transfected with WT or SNP-PARP1 vectors. Twenty-four hours post transfection, cells were divided to 96-wells and were incubated with DMSO or 10 nM Olaparib for additional 48 h. Proliferation rate was assessed at the presence of EZF4 reagent. Spectrophotometer results were normalized. Olaparib treatment was normalized to DMSO, while bars represent the fold stimulation of sh-BRCA1 against values obtained from sh-control cells. The value “1” demonstrates that there is no difference in the proliferation rate of sh-BRCA1 versus sh-con while WT-PARP1 was introduced. Upon SNP-PARP1 transfection, sh-BRCA1 cells become less proliferative under Olaparib treatment (*p value < 0.05).

PARP inhibition requires DNA DSB repair, which can be monitored by assessing the amount of the phosphorylated form of the histone H2A variant, γ-H2AX, which forms rapidly at the sites of DNA DSBs^34,35^. We incubated two ovarian cell lines, SKOV3 and Heya8 (WT-PARP1 and SNP-PARP1, respectively), with 10 µM Olaparib for 2 h, at 37 °C and visualized the typical foci-like pattern produced, using a florescence microscope (Fig. 5a). MOCK cells were used as a control. The cell profiler tool (the CellProfiler project team is based in the Carpenter Lab at the Broad Institute of Harvard and MIT) was used to count the number of foci in each cell line with and without Olaparib in the incubation mix. The results in Fig. 5b demonstrated that the WT-PARP1 Heya8 cells are hypersensitive to Olaparib treatment with mean foci evaluated at 35.22, compared to the value of 20.31 in the MOCK (control) cells.

The SKOV3 cell line demonstrated a mild increase in mean foci, after Olaparip treatment (increase from 18.65 to 23.8, post treatment) although the 1.734-fold increase in mean foci in the Heya8 cells was significantly higher than the change in the SKOV3 cell line foci after Olaparib treatment (1.27). Taken together, the results obtained by measuring the phosphorylated form of γ-H2AX confirmed our previous observations that the SNP version of PARP1 is more sensitive to Olaparib than the WT-PARP1.

The high negative charge of the PAR polymers leads to dissociation from DNA, which is a required step for DNA repair completion. In the presence of a PARP inhibitor, however, PARylation is inhibited by PARP1 activity trapping^36^. Since de-PARylation is at least partly based on allosteric interactions, it was of interest to consider any SNP-related structural variations among PARP1 variants. For this reason, we next assessed the PARylation levels of PARP1-GFP variants, with or without PARP1i. The rationale was that comparing the behavior of MOCK WT versus SNP-PARP1 GFP will reflect the differences in the native pattern of PARylation due to local conformation variations at the PAR-binding domain of PARP1 variants. In contrast, differences in the PARylation profiles of variants treated with Olaparib will reflect the importance of PARP1 conformation in the response to Olaparib therapy.

HEK293 lines stably expressing WT-PARP1-GFP or SNP-PARP1-GFP were treated with increasing doses of Olaparib (0, 0.01, and 0.1 µM) for 30′ at 37 °C. The results presented in Fig. 5c indicated a higher expression of SNP-PARP1-GFP (higher band) than the WT-PARP1-GFP variant (preserving equal endogenous PARP1 levels). However, interestingly, re-blotting the membrane with anti-PAR antibody revealed that WT-PARP1-GFP was PARylated to a greater extent than the SNP variant.

The next challenge was to identify the contribution of BRCA1 silencing to the PARP1 variant-dependent-response. This concept is based on the mechanism of action (MOA) of Olaparib, which, as already mentioned in the Introduction, involves synthetic lethality. We have already generated a stable system which silences the endogenous BRCA1 using a specific short hairpin vector (sh-BRCA1 or sh-Control) in HEK293T cells under puromycin selection (Supplementary Fig. 4). PARP1 variants were introduced into these cell lines and they were treated with Olaparib to examine the effect of a PARP1i on their proliferation rate (compared to DMSO as control). Proliferation was measured by the EZ4U cell proliferation and cytotoxicity assay, which is based on the capability of living cells to reduce slightly colored or uncolored tetrazolium salts in the mitochondria to form intensely colored formazan derivates. This water-soluble formazan is secreted into the culture medium and can be measured with a standard colorimetric reader. The absorption values obtained at 450 nm were normalized against those obtained at 620 nm, according to the manufacturer’s protocol. Results are presented as the relative proliferation ratio (sh-BRCA1/sh-Control) for each PARP1 variant. As can be seen in Fig. 5d, treatment with Olaparib reduced the rate of proliferation of SNP-PARP1 cells by ~25% when BRCA1 was silenced but had no effect on the relative proliferation rate in WT-PARP1 sh-BRCA1 cells (sh-BRCA1/sh-Control ratio of 1).

## Discussion

This study was designed to test the hypothesis that a silent, population prevalent, difference in coding sequence (SNP) may lead to structural variation in the coded protein^37^. We attribute these structural variations to changes in the ribosome translation rate, which is regulated at least partly, by tRNA reserves and by mRNA secondary structure. Our results lead us to recommend that the SNP status of patients should be involved in decision making when considering treatment options with PARPi-derived drugs.

The linear nucleotide composition of mRNA is known to influence the secondary structure. By using the tools mFOLD and RNAsnp, we were able to demonstrate that a single-nucleotide substitution at the specific position, Chr 1: 226385663 (GRCh38), is responsible for both global and local changes to the WT version of the human PARP1 gene (Fig. 1c, d). mRNA transcripts with relatively low secondary structure complexity will associate faster with the small ribosomal subunit (40S), and therefore will be more likely to form stable initiation complexes, which is a critical event in determining translation efficiency^38^. Moreover, SNPs require the use of specific tRNAs, which may differ from those used for the wild-type version with the result that while certain codons for the same amino acid benefit from availability of tRNAs, other codons experience tRNA shortage. Lack of tRNA may result in translational delay. Under these constraints, the newly synthesized peptide may acquire a new thermodynamic equilibrium to maximize stability. These micro-adaptations may expose or hide functional sites of the mature protein and alter its function.

We have demonstrated that rs1805414, a human PARP1-SNP, is associated with higher levels of mRNA levels than the WT or heterogeneous genotype, in a cohort of 415 breast cancer patients from the TCGA. These results were further supported by direct PARP1 mRNA measurements in SKOV3 (WT/WT PARP1 genotype) compared to COV362 (SNP/SNP PARP1 genotype) ovarian cell lines. We speculate that the variation in mRNA levels, across WT and SNP variants (Fig. 1b, e), may be attributed to both changes in the secondary mRNA structure and availability of the tRNA pools which, alone or together, determine the translation rate. The obtained mRNA levels reflect the ribosome “occupied” mRNA fraction.

As shown in Fig. 2a, the mRNA levels of HEK293T cells over expressing GFP-WT-or-SNP PARP1 displayed the same trend as endogenous mRNA (Fig. 1). However, an inverse trend was observed when we extracted protein and compared the expression levels of GFP-PARP1 WT and SNP variants to endogenous PARP1. This trend was further supported by analysis of the SKOV3 and COV362 cell lines, which represent endogenous WT and SNP PARP1, respectively. While the levels of WT PARP1 mRNA were relatively high in comparison to the SNP version, the opposite trend was seen for the protein levels. This goes against the assumption of a direct correlation between mRNA and protein levels and could be interpreted by the streamlined nature of the translation. If the occupied mRNA fraction (by ribosomes) holds the newly synthesized peptide, low levels of free mRNA will be associated with high levels of protein levels as was demonstrated both endo/exogenously. This hypothesis was tested by simulating the dynamics of translation using the delayed Stochastic Simulation Algorithm (delayed SSA)^26^. This allowed us to follow the biological process computationally and demonstrate that variability in the SNP reaction rate gives rise to a considerable difference in the average amount of protein produced. These results are in agreement with experimentation.

We hypothesize that SNP dictated translation is accelerated due to high availability of tRNA, while translation of the WT PARP1 is delayed by tRNA shortage. As mentioned above, such a delay may account for the introduction of micro-structural aberrations into the mature protein. We could detect such changes by comparing the chymotrypsin and trypsin, cleavage patterns generated from WT or SNP PARP1-GFP proteins. While the trypsin cleavage profile was the same across PARP1 GFP variants, the chymotrypsin cleavage pattern was variant specific: SNP-PARP1 GFP cleavage produced a 25 kDa product whereas the WT-PARP1 GFP substrate was cleaved to give three by-products, of 60, 50, and 25 kDa. All products were tracked by western blot, thanks to the C′ terminal GFP tag of each PARP1 GFP variant. The difference in digestion pattern may be explained by structural variation in the mature protein, which affected the proteolytic enzyme accessibility.

Since the results implicated SNP as a leading cause of protein variation, we investigated the presence of any SNP-related post-translation modifications. Specifically, we traced the ubiquitination status of PARP1 GFP proteins, in the context of overexpressed HA-UBB, a tagged version of ubiquitin. Indeed, we could characterize two ubiquitination patterns: while the WT PARP1 GFP demonstrated a typical smear of ubiquitination, and a relative intense expression level, the SNP PARP1 GFP was free of smear with a relatively low level of expression. This could be attributed to accessibility issues, between WT and SNP PARP1 GFP that may permit SNP PARP1 GFP free access to the UBB-HA and consequent degradation, while the WT PARP1 GFP remains attached to the UBB-HA, and degradation is delayed. An alternative explanation is that because of the slower rate of translation, the post-translation modification of WT PARP1 GFP (via UBB-HA) is delayed compared to the SNP variant. Interestingly, overexpression of an empty vector preserved the expression level trend across PARP1 variants. Together, we conclude that the results indicate that SNP involvement in protein architecture may lead to functional differences in the mature protein.

Structural variations across PARP1 variants may also be detected by measuring Wnt signaling, which is a key step during embryonic development, tissue homeostasis, and regeneration^29^. In order to study Wnt signaling in the context of these specific changes to PARP1, we used the TOP-Flash system, a construct containing three copies of a wild-type TCF-4-binding element (pTOPFLASH), which allowed us to measure the Wnt signaling rate, while introducing PARP1 variants, one at a time. The results identified PARP1 as one of the co-activators of the Wnt pathway through β-catenin-TCF-4 activation complex^29^. PARP1 is cleaved by caspase-3 during the course of apoptosis^28^. This cleavage is likely to inhibit the interaction between PARP1 and TCF-4 (ref. ^29^) and finally leads to Wnt signaling termination. Our results indicated that SNP PARP1 increased luciferase activity over the WT PARP1 variant (Fig. 3c). This was supported by western blot analysis, demonstrating that the SNP PARP1 GFP variant was overexpressed compared to relatively low expression of the corresponding WT PARP1 variant. This phenomenon could have three alternative mechanistic explanations: (a) WT and SNP PARP1 variants differ in their caspase-3-binding domain, (b) the PARP1 variants differ in their binding domain to the TCF-4/β-catenin complex, or (c) the autopolyADP-ribosylation sites on PARP1 variants are structurally different and force a variation in Wnt signaling levels.

The conformational changed associated with SNP status may be of clinical relevance. As demonstrated by the ubiquitination assay and Wnt signaling system, WT PARP1 differs from the SNP version at the protein level. In order to learn more about the correlation between PARP1 status and response to PARP1i, we analyzed two public data sets, GDSC and CTD2, which contain sets of data concerning the responses of cell lines to an array of drugs. For our purposes, we selected Olaparib and Veliparib (ABT-888), two commonly used PARP1i drugs. Based on the whole-exome sequencing of the CCLE, we were able to identify the PARP1 status of each recorded cell line and then consider the response to each of these drugs, through their measured AUCs.

The results from both data sets (Fig. 4b, c) were in general agreement that Olaparib is more effective in cell lines with the SNP PARP1 variant than those with the WT version of the PARP1 (Veliparib followed the same trend but the difference was statistically insignificant). These results support what we have seen in the Biacore assay where, while both PARP1 variants had relative high affinity to Olaparib, the SNP PARP1 variant had a fivefold lower *K*_D_ (Fig. 4d), than the WT, suggesting higher binding affinity to Olaparib. This led us to the possibility of a clinical implication: patients should be classified prior to treatment with Olaparib (or any PARP1i) according to the PARP sequence. The SNP PARP1, though presenting minor structural differences compared to the WT version, shows higher binding affinity to Olaparib. This would result in a correlation between patients’ PARP1 status and treatment outcome/response. It appears that SNP PARP1 carriers may benefit more from Olaparib treatment than homozygous WT PARP1 subjects.

To explore the DSB repair aspect, we measured γ-H2AX, the phosphorylated form of H2A histone family member X (H2AX), as a way to monitor the DSB repair rate. The ratio of DNA DSBs to visible γ-H2AX foci is close to 1:1, which forms the basis of a sensitive quantitative method for detection of DNA DSBs in mammalian cells^39,40^. The higher ratio observed between treated and untreated Heya8 cells (SNP PARP1 variant) compared to treated to untreated SKOV3 cells (WT PARP1) demonstrated that the SNP PARP1 genotype is much more sensitive to Olaparib than the WT PARP1 form. The response to Olaparib is not trivial and could be multifactorial. Nevertheless, the most critical factor in the tested cells is the genomic status of PARP1. Since the Olaparib mechanism of action involves a direct binding of the drug to the PARP1, potential changes in protein structure may dictate the level of drug interaction. This concept was previously suggested by Kimchi-Sarfat et al.^13^ and may constitute a promising strategy in personalized medicine, where patient genotype is the main argument/consideration for the drug decision process.

To better support our line of thinking, we examined another measurable phenomenon associated with PARP1i treatment. PARP1 inhibitors were thought to enhance catalytic inhibition, until Murai and colleagues^30^ revealed that, they effectively “trap” the damaged DNA in cytotoxic PARP1–DNA complexes.

The association of PARP1 with damaged DNA depends on PARP1 PARylation status where only the non-PARylated fraction of PARP1 is able to interact with the damaged DNA. PARP1 inhibitors promote the initiation of such cytotoxic complexes by reducing the auto-PARylation levels. In this context, allosteric issues concerning PARP1 proteins may be of great importance when PARP1 inhibitors are involved, since PARP1i initiates the cascade through direct binding to its target, PARP1. We used the fact that the PARylation status of PARP1 variants can be easily detected by western blot analysis. Overexpressed PARP1 GFP variants were assessed for their expression levels and compared to endogenous PARP1. Next, by employing anti-PAR antibody, we were able to demonstrate large differences in the PARylated PARP1 fraction, between variants. WT PARP1 GFP, though relatively poorly expressed in comparison to its SNP version, exhibited high levels of PARylayion, when blotted with anti-PAR (Fig. 5c). This observation might be of importance in the context of PARP1i treatment. As already mentioned, the relatively basal high PARylation levels of WT PARP1 impose a challenging microenvironment for the PARP1i, which partly act to promote de-PARylation. Such a scenario would lead PARP1i to favor the SNP PARP1 variant over the WT version, and actually make SNP PARP1 more sensitive to PARP1i treatment.

The design of many therapeutic agents involves customizing the drug to interact with a specific protein. Knowledge of the structure of the desired protein can be obtained from the Protein Data Bank archive (PDB), which is a repository of information of the 3D structures of proteins, nucleic acids, and complex assemblies^41^. These 3D structures are usually obtained by crystalizing the protein of interest in artificial systems that involve in vitro translation, and which by definition, do not consider tRNA usage. Such cell-free systems may bias the configuration of the studied protein and thus expose or hide specific protein sites, which are relevant under physiological conditions.

To relate our results to the clinical condition, we included the BRCA1 mutation as a supportive criterion in determining drug response based on the Olaparib synthetic lethality MOA. By employing the sh-BRCA1 construct, we were able to demonstrate that BRCA1 silencing reduced the proliferation rate of Olaparib treated cells to a greater degree if they harbored the SNP-PARP1 rather than the WT version (Fig. 5d). This oversensitivity to Olaparib shown by the SNP-PARP1 variant under BRCA1 silencing provides additional support for our SNP dependent protein alternation argument.

We believe these results suggest that PARP1 status might lead to downstream changes to protein structure. A needed next step to translate these results to the clinic could be the evaluation of clinical samples from patients once they have been treated with first-line Olaprib. These samples should be evaluated for their PARP1 status together with their BRCA1 status.

## Methods

### The Cancer Genome Atlas (TCGA)

Data were obtained from The Cancer Genome Atlas (TCGA) database http://cancergenome.nih.gov/. This dataset comprises of whole-exome sequencing, RNA sequencing, and clinical information for 415 Breast Cancer (BRCA) patients.

### In silico RNA analysis

The RNA structure of all the variants was predicted with the RNA Mfold program at http://www.bioinfo.rpi.edu/applications/mfold. Local change significance was calculated using RNAsnp https://rth.dk/resources/rnasnp/ for the calculation of the changes *p* value.

### Cell lines

HEK293T cells were purchased from ATCC. Cells were grown at 37 °C with 5% CO_2_ in DMEM medium supplement with 2 mM l-glutamine, 1.5 g/l sodium bicarbonate, 4.5 g/l glucose, 10 mM HEPES, 1.0 mM sodium pyruvate, and fetal bovine serum (FBS).

Generation of stable 293T-shBRCA1 cells—293T cells were transfected with 2 µg of sh-BRCA1 GFP or CON. Twenty-four hours post transfections, cells were treated with 2 µg/ml Puromycin (Invitogen, #ant-pr) for 2 weeks to obtain stable colonies. The positive colonies were screened based on their GFP expression as visualized by a florescence microscope. Constructs were a generous gift from Dr. Ra’anan Berger, Sheba Medical Center.

HeyA8 ovarian cell lines (obtained as a generous gift from Prof. Shay Israeli) were grown at 37 °C with 5% CO_2_ in MEM-EAGLE medium supplement with 2 mM l-glutamine, 1.5 g/l sodium bicarbonate, 4.5 g/l glucose, 10 mM HEPES, 1.0 mM sodium pyruvate, and 10% FBS.

COV362 ovarian cell lines were grown at 37 °C with 5% CO_2_ in DMEM medium supplement with 10% FBS and Pen/sterp.

SKOV3 ovarian cell lines were grown at 37 °C with 5% CO_2_ in McCoy’s 5A medium supplement with 10% FBS, 1% Pen–strep, 0.1 mM nonessential AA, and 2 mM l-glutamine.

### Simulation

In this section we describe the stochastic model developed for comparing the translating process of a WT vs. SNP-mRNA. The main idea behind our formulation is to separate the translation process to pre/post-SNP event, such that we can focus on the contribution of the SNP alone. More specifically, we denote the process of translating the first part as mRNA1, which is responsible for generating the p1 part of the protein. This is followed by the SNP codon translation that presumably differs between the two cases, then a similar process to the first one, but now for the second part of the mRNA2. Finally, a single such process concludes with releasing the mRNA and ribosome, in addition to the generated protein. Each step of the whole process is modeled by a reaction equation with control parameters, rate and delay. To simplify the model, we do not model explicitly the translation of each codon in mRNA1 and mRNA2, but the whole sub-process with a proper delay that takes into account the length of each sequence.

Equation (1) models the first translation reaction, taking a mRNA and a ribosome that create a complex with a rate *k*_*p*1_, and translating mRNA1 until the SNP codon with a time delay *τ*_*p*1_. Equation (2) describes the translation of the SNP (or WT, depending on the parameters), taking a tRNA that binds the codon with a rate *k*_snp_, translating it and releasing the tRNA after a delay-*τ*_snp_. Equation (3) is similar to Eq. (1), but with a delay *τ*_*p2*_ reflecting the possible length difference. Equation (4) is the final stage—releasing the components, each with a different delay. Equation (5) describes the protein degradation process with a rate- $k_{P\_{\mathrm {decay}}}$. We stress that p1, mRNA1, and mRNA2 are in fact dummy molecules, and serve only for simulation purpose, i.e., they are not actual free molecules, but rather they are artificially labeled to facilitate the focus on the codon-of-interest. This constraint is modeled by the complexes, e.g., p1.Rib.mRNA1, which means that once the mRNA and ribosome creates a complex, they are not to be considered separately.

The parameters taken in the simulation are as given below (time delays units are s, and rates are 1/s). The parameters are based on the review^26^, and were chosen as follows:

$$\begin{array}{l}k_{p1} = 0.01,\,k_{\mathrm {snp}} = 1e^{ - 3} \ast \left[ {0.4,0.8,1.6,4} \right],\,k_{P\_{\mathrm {decay}}} = 3e^{ - 4},\\ \tau _{p1} = 10,\,\tau _{p2} = 25,\,\tau _{\mathrm {snp}} = [0.1,1,5],\,\tau _{\mathrm {mRNA}} = 5,\,\tau _{\mathrm {Rib}} = 50,\,\tau _{\mathrm {prot}} = 300.\end{array}$$

Initially, the amount of molecules was set to: Rib = 100, mRNA = 50, tRNA = 50. These numbers are fixed throughout the simulation, since we do not model the transcription process, that is, no new mRNAs are created, and each one molecule that is released (Eq. 4), returns to the pull. Same is true for the ribosomes and tRNA. The amount proteins is initialized to Prot = 0; however, this value changes dynamically as a result of creation and decay.

1 $${\mathrm {Rib}} + {\mathrm {mRNA}}_{1}\mathop { \to }\limits^{k_{p1}} {\mathrm{p1}}.{\mathrm{Rib}}.{\mathrm{mRNA}}_{1}\left( {\tau _{p1}} \right),$$

2 $$\begin{array}{l}{\mathrm {p1.Rib.mRNA}}_{1} + {\mathrm {tRNA}}\mathop {\to}\limits^{k_{\mathrm {snp}}} {\mathrm {snp.p1.Rib.mRNA}}_{1}\left( {\tau _{\mathrm {snp}}} \right)\\ +\, {\mathrm {tRNA}}\left( {\tau _{\mathrm {snp}}} \right),\end{array}$$

3 $${\mathrm {snp.p1.Rib.mRNA}}_{1} + {\mathrm {mRNA}}_{2} \mathop { \to }\limits^{k_{p1}} {\mathrm {mRNA}}_{2}{\mathrm{.snp.p1.Rib.mRNA}}_{1}\left( {\tau _{p2}} \right),$$

4 $$\begin{array}{l}{\mathrm{mRNA}}_{2}{\mathrm{.snp.p1.Rib.mRNA}}_{1} \mathop { \to }\limits^1 {\mathrm {mRNA}}_{1}\left( {\tau _{\mathrm {mRNA}}} \right) + {\mathrm {mRNA}}_{2}\left( {\tau _{\mathrm {mRNA}}} \right)\\ +\, \mathrm {Rib}\left( {\tau _{\mathrm {Rib}}} \right) + \mathrm {Prot}\left( {\tau _{\mathrm {prot}}} \right),\end{array}$$

5 $${\mathrm {Prot}}\mathop { \to }\limits^{k_{P\_{\mathrm {decay}}}} \emptyset.$$

### Construction of GFP-PARP1 variants

GFP-PARP1 variants obtained by introducing a point mutation at the following locations: +852T>C, +852T>G using an appropriate primer set and the QuickChange II Site-Directed Mutagenesis Kit (Agilent Technologies) on the GFP-PARP1 template, which was a generous gift from Prof. John Pascal.

### RNA extraction and cDNA synthesis

Total RNA was isolated from the cultured cells following transfection using Tri reagent (Ambion® Life Technologies Co. GI, NY, USA) according to the manufacturer’s instructions. Up to 1 μg of RNA was used for cDNA synthesis using High Capacity cDNA Reverse Transcription kit (Applied Biosystems, Life technologies Co.) according to the manufacturer's protocol.

### Real-time qPCR

Real-time qPCR reactions are performed to quantitate genes expression. Each PCR reaction contains 2 μl of serially diluted cDNA samples, 10 pmol of each forward and reverse primer, complementary to the tested gene or β-actin, as a loading control, and 10 μl KAPA Syber FAST ABI Prism qPCR Kit (Kapa Biosystems Inc. Woburn, MA, USA). Reactions were run on 7900HT Real-Time PCR (Applied Biosystems, Life technologies Co.) instrument in FAST mode with standard curve program keeping the manufacturer defaults. The primers sequences are as follows:

GFP: Forward: 5′-TCTGTCTCCGGTGAAGGTGAAG-3′

Reverse: 5′-GGCATGGCAGACTTGAAAAAG 3′.

PARP1: Forward: 5′-GCCGAGATCATCAGGAAGTATG-3′

Reverse: 5′-ATTCGCCTTCACGCTCTATC-3′.

β-actin: Forward: 5′-AGCGAGCATCCCCCAAAGTT-3′

Reverse: 5′-GGGCACGAAGGCTCATCATT-3′.

BRCA1: Forward: 5′-ACAGCTGTGTGGTGCTTCTGTG-3′

Reverse: 5′-CATTGTCCTCTGTCCAGGCATC-3′.

### Western blotting

Cell extracts were prepared with cell lysis buffer (1% Triton X-100, 150 mM NaCl, 50 mM Tris HCl, pH 7.4, 1 mM EDTA, and protein inhibitor cocktail (Sigma Aldrich Co. Rehovot, Israel)) on ice, 48 h after transfection. Following SDS polyacrylamide gel electrophoresis (SDS-PAGE) separation, proteins were transferred to nitrocellulose membranes and blocked with 5% low-fat milk. Membranes were incubated with rabbit anti-GFP, anti-PARP1, or anti-Actin primary antibodies, washed with PBS containing 0.001% Tween-20 (PBST) and incubated with the appropriate horseradish peroxidase-conjugated secondary antibody, Goat anti-rabbit-HRP, or anti-mouse-HRP. After washing in PBST, membranes were subjected to enhanced chemiluminescence detection analysis.

### Statistical analysis

Student’s *t-*tests were used for statistical analysis of differences among samples.

### Chymotrypsin cleavage

Protein extracts were diluted to a protein concentration of 2.5 µg/µl. Five hundred microliters extracts were treated with Chymotrypsin (final concentration 0.5 µg/ml) at 37°. A 100 µl sample was taken from the reaction at each time point as indicated in the text. Protein samples were mixed with protein loading buffer and resolved by SDS-PAGE.

### Ubiquitination assay

PARP1 WT or SNP-GFP was transiently co-transfected to HEK293T cells along with UBB-HA or empty vector. Forty-eight hours later, cells were harvested and subjected to SDS-PAGE gel. By using anti-GFP antibody we detected the overexpressed GFP-PARP1 of both the WT version and the SNP variant. The un-bounded UBB-HA was detected by anti-HA antibody.

### WNT signaling

For Wnt signaling reporter assays, HEK293T cells growing in 24-well dishes were transfected at 60–70% confluence with 2 μg GFP-PARP1 WT or SNP, 0.5 μg pTOPFLASH (reporter plasmid), and 0.1 μg β-gal. Forty-eight hours after transfection, cells were solubilized in reporter lysis buffer (Promega) and luciferase levels were measured. β-Gal was used to monitor transfection efficiency.

### γ-H2AX

We incubated each cell line with 10 µM Olaparib for 2 h, at 37 °C compared with MOCK cells. Cells were then fixed, washed with PBS-Triton X-100 buffer to allow anti-γ-H2AX (anti-gamma H2AX (phospho S139) antibody (Abcam) permeabilization and incubated with a secondary anti-FITC goat anti-rabbit secondary antibody (ab6717), DAPI stained and mounted (UltraCruzTM Mounting Medium, Santa Cruz). We visualized the typical foci-like pattern by the florescence microscope, Leica confocal live, at ×60 magnitude. Using cell profiler tool (The CellProfiler project team is based in the Carpenter Lab at the Broad Institute of Harvard and MIT) we counted the number of foci in each cell line under no treatment (basal phospho-H2AX)−MOCK and under Olaparib treatment.

### Drug response data

Olaparib and Veliparib response was measured in two different cell line datasets—CTD2 and GDSC. For each cell line the AUC value was measured, and a *Z*-score was calculated. The cell lines were separated according to their PARP1 status from the CCLE.

### Biacore

HEK293T cells were transfected with GFP-PARP1 WT or SNP. Forty-eight hours later, cells were lysed in HEPES Biacore buffer (10 mM HEPES, 150 mM NaCl, 3 mM EDTA, 0.05% TWEEN-20) and protein inhibitor cocktail (Sigma Aldrich Co). Series S Sensor chip CM5 single (GE) was pre-treated with Trap-GFP antibody (ChromoTek GmbH, gt-250). Each lysate was streamed through the different chip channels, attached by the Trap-GFP antibody and further incubated with 13 serial dilutions of PARP1 inhibitor Olaparib (LKT-O4402) (starting con. 250 nM). The sensogram for the drug is presented, as obtained from Bicore T100 analysis.

### Parylation

HEK293 stably expressed WT-PARP1-GFP or SNP-PARP1-GFP were treated with elevated doses of Olaparib (0, 0.01, and 0.1 µM) for 30′ at 37 °C. Lysates were subjected to SDS-PAGE gel and the blotted membrane was incubated with anti-PARP1 rabbit antibody (H-250) (sc-7150) (Santa Cruz) and with anti-GAPDH rabbit (ab8245). Secondary Peroxidase Affinity Pure Goat Anti-Rabbit IgG (H+L) (111-035-144; Jackson ImmunoResearch) was used soon after.

### Proliferation assay

2 × 10^7^ cells of each HEK293 stable cell line (sh-BRCA1 or sh-Control) were transfected with WT- or SNP-PARP1 GFP (2 μg per single six well). Twenty-four hours later, cells were resuspanded and further divided to 96-wells plate (1 × 10^4^ cells/100 µl/well). We enabled cells to re-adhered and incubated cells in the presence of DMSO or Olaparib (10 µM concentration). Post 48 h incubation with the drug, we add 20 µl of EZ4U reagent (Biomedica, cat # BI-5000) and incubated for additional 4 h at 37 °C. By using an Elisa reader at wave length of 450 and 620 nm, according to the manufacturer's protocol, we were able to monitor relative cell proliferation per each PARP1 variant, as a result from its behavior in sh-BRCA1 condition versus sh-control. The EZ4U cell proliferation and cytotoxicity assay is based on the capability of living cells to reduce slightly colored or uncolored tetrazolium salts in the mitochondria into intensely colored formazan derivates. This water-soluble formazan is secreted into the culture medium and can be measured with a standard colorimetric reader.

### Reporting summary

Further information on research design is available in the Nature Research Reporting Summary linked to this article.

## Supplementary information

Reporting Summary

Supplemental figures S1,S2,S3,S4,S5

## Data Availability

The datasets generated during and/or analyzed during the current study are available in the TCGA repository, https://cancergenome.nih.gov/. *Cell line databases*: GDSC and CTD2 are available at https://www.cancerrxgene.org/ and https://ctd2-dashboard.nci.nih.gov/dashboard/, respectively.
