# Supplementary material for: A single nucleotide variant of human PARP1 determines response to PARP inhibitors
Source: NPJ Precis Oncol. 2020 Apr 27;4:10. doi: 10.1038/s41698-020-0113-2 (PMC7184601; doi:10.1038/s41698-020-0113-2)
Supplement: Supplementary file 2 — Supplemental figures S1,S2,S3,S4,S5 [file 41698_2020_113_MOESM2_ESM.pdf]

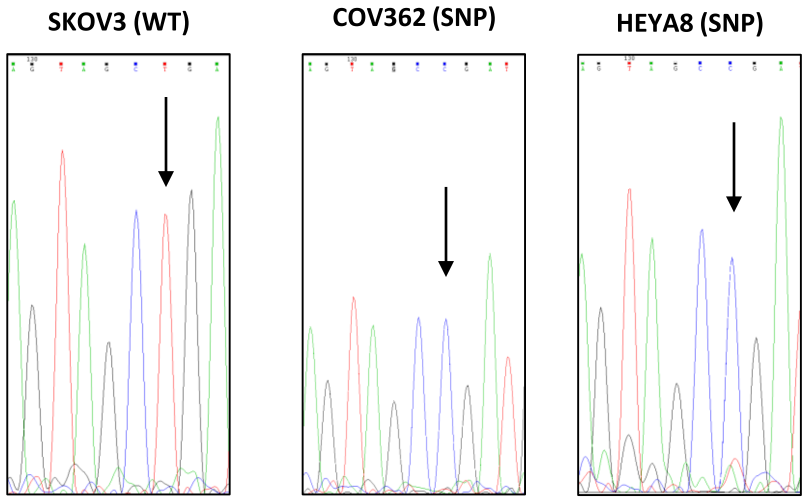

Supplementary Figure 1

Supplementary Figure 1. Sanger sequencing was employed to verify PARP1 status of SKOV3, COV362 and Heya8 Ovarian cell lines, by using the indicated primers (in the Methods section), which surround the SNP position from both sides.

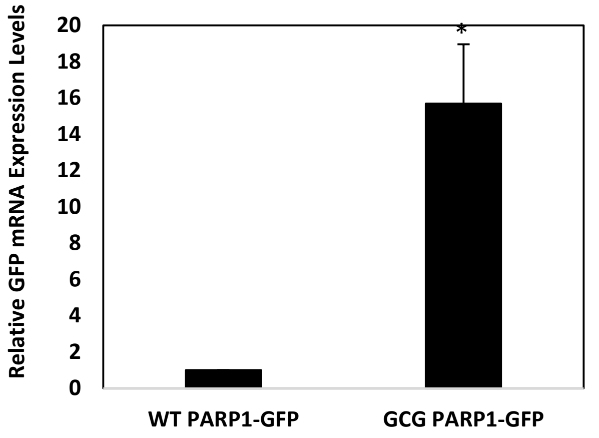

Supplementary Figure 2

Supplementary Figure 2. We introduced a GCG artificial variant of PARP1 to the WT-PARP1 vector by SDM manipulation. GCG-PARP1-GFP and WT-(GCT)-PARP1-GFP plasmids were over expressed in HEK293T cells. 48h later, mRNA levels of these PARP1 variants, demonstrated **high** levels of PARP1 mRNA compare to WT PARP1 GFP (p Value < 0.05).

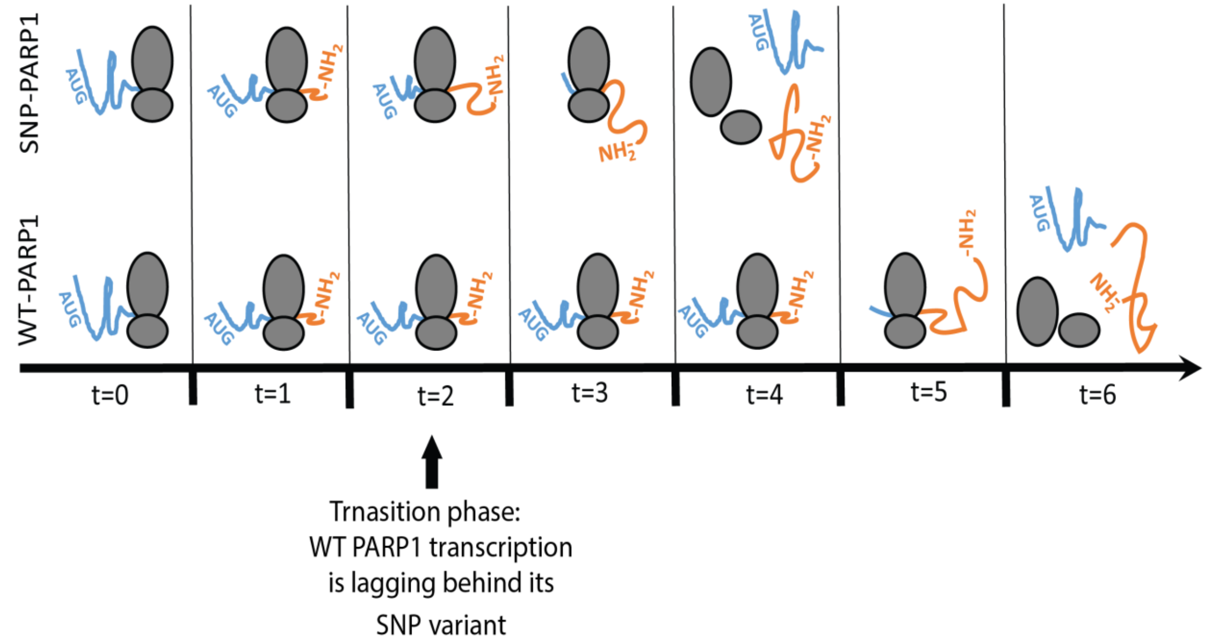

Supplementary Figure 3

Supplementary Figure 3. A schematic illustration of translation. SNP versus WT –PARP1 mRNAs are being processed though ribosomes machinery to obtained the mature protein. A lagging phase in WT-PARP1 translation, contributed by lack of an appropriate tRNA, result in delay on ribosome and rearrangement of the newly synthesis peptide. While the SNP-PARP1 mRNA complicated an entire cycle of translation, ready to initiate an additional cycle, the WT-PARP1 repairing its tRNA and continued a first cycle, performing new protein structure compare to its SNP-PARP1 version.

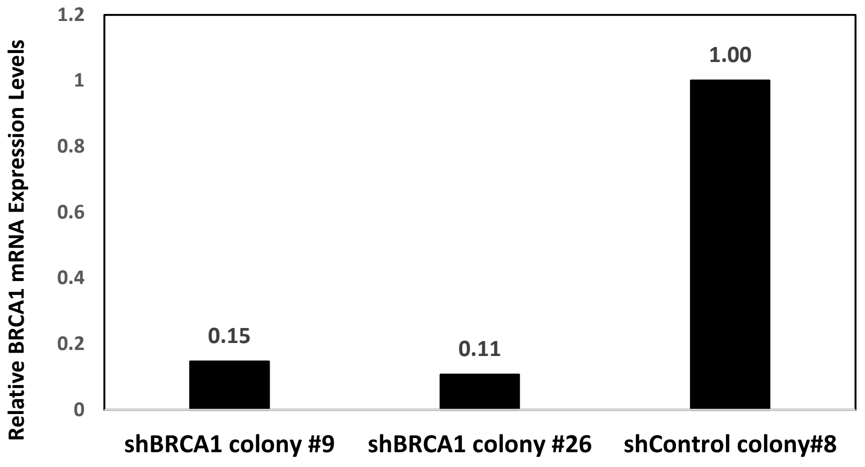

Supplementary Figure 4

Supplementary Figure 4. HEK293T cells were transfected with 2 μg of a specific short hairpin vector, sh-BRCA1 or sh-Control to generate a stable system of silenced endogenous BRCA1. 48h post transfection, cells were exposed to 2μg/μl puromycin selection, until stable colonies were generated. RNA was extracted from the stable cells and mRNA levels of BRCA1 were analyzed by qRT-PCR, normalized to β-actin (primers are indicated in the Methods section).
